# Significance of Premature Vertebral Mineralization in Zebrafish Models in Mechanistic and Pharmaceutical Research on Hereditary Multisystem Diseases

**DOI:** 10.3390/biom13111621

**Published:** 2023-11-06

**Authors:** Judith Van Wynsberghe, Olivier M. Vanakker

**Affiliations:** 1 Center for Medical Genetics, Ghent University Hospital, 9000 Ghent, Belgium; judith.vanwynsberghe@ugent.be; 2 Department of Biomolecular Medicine, Ghent University, 9000 Ghent, Belgium; 3 Ectopic Mineralization Research Group, 9000 Ghent, Belgium

**Keywords:** zebrafish model, multisystemic disorders, premature mineralization, axial skeleton

## Abstract

Zebrafish are increasingly becoming an important model organism for studying the pathophysiological mechanisms of human diseases and investigating how these mechanisms can be effectively targeted using compounds that may open avenues to novel treatments for patients. The zebrafish skeleton has been particularly instrumental in modeling bone diseases as—contrary to other model organisms—the lower load on the skeleton of an aquatic animal enables mutants to survive to early adulthood. In this respect, the axial skeletons of zebrafish have been a good read-out for congenital spinal deformities such as scoliosis and degenerative disorders such as osteoporosis and osteoarthritis, in which aberrant mineralization in humans is reflected in the respective zebrafish models. Interestingly, there have been several reports of hereditary multisystemic diseases that do not affect the vertebral column in human patients, while the corresponding zebrafish models systematically show anomalies in mineralization and morphology of the spine as their leading or, in some cases, only phenotype. In this review, we describe such examples, highlighting the underlying mechanisms, the already-used or potential power of these models to help us understand and amend the mineralization process, and the outstanding questions on how and why this specific axial type of aberrant mineralization occurs in these disease models.

## 1. Introduction

Over the last 20 years, zebrafish have become an important model organism for studying the development and diseases of the skeleton in basic and preclinical research [1,2]. In general, the popularity of zebrafish as a model system is associated with their rapid external development, short generation time, large offspring number (which increases statistical power due to higher sample numbers), and imaging possibilities that are facilitated by the transparency of zebrafish embryos and larvae [1,2,3,4,5]. Genetically, there is 71% homology between the human and zebrafish genome, with 82% of the human-disease-causing genes having a zebrafish orthologue [3,6]. This is also true for key bone development regulators, which are highly conserved and have similar expression patterns in zebrafish and humans [2,7]. An additional advantage compared to mouse models, wherein many mutants with bone defects are embryonic lethal or die soon after birth, is that zebrafish mutants usually survive to early adulthood, possibly because of the reduced load on the skeleton in aquatic animals [2,8].

The axial skeleton of the zebrafish has been put forward as a model for understanding common spinal deformities, such as (idiopathic) scoliosis, and degenerative diseases like osteoarthritis and osteoporosis [9]. To interpret the effects of gene mutations and expression patterns and establish correlations between genes and morphology, a comprehensive understanding of the development of the axial skeleton is necessary. Methods such as systematic genome-wide mutagenesis screens can be used to understand the early patterning of the embryonic tissue required to build and pattern the embryonic spine [10]. Forward screens using N-ethyl-N-nitrosourea (ENU) have increased our knowledge on the postembryonic maturation and homeostasis of the spine, but the involved mechanisms remain poorly understood [10,11,12,13,14,15,16].

For many multisystem disorders, that is, diseases that affect multiple body systems, zebrafish have been instrumental in understanding the pathophysiological mechanisms of disease and the preclinical evaluation of the effects of drugs on one or more affected organ systems. Further, the partitioning of the expression patterns and functions of the duplicated zebrafish genome can be particularly helpful in untangling the different (patho)physiological roles of a gene [17,18,19]. Interestingly, several zebrafish models for hereditary multisystem disorders that do not affect the vertebral column in humans are characterized by abnormalities in the mineralization and morphology of the axial skeleton. In this review, we provide an overview of such zebrafish models, including the involved genes and signaling pathways. We illustrate how the axial skeleton can be a valuable read-out for the modeling of and therapeutic intervention in complex multisystemic disorders and how these disease models can be useful for providing insights into the biology of normal and pathological (skeletal) development in zebrafish. 

## 2. Materials and Methods

A literature search was conducted via the Pubmed, ScienceDirect, Embase, and Google Scholar databases, limited to papers published in English and using the following key words: ‘zebrafish’, ‘disease model’, ‘axial skeleton’, ‘vertebral column’, ‘vertebra’, ‘mineralization’, and ‘calcification’. Exclusion criteria included papers on a bone disease or on a vertebral deformation disease (e.g., scoliosis). The remaining papers were further reviewed to ascertain whether the modeled disease was a multisystemic disorder. For each of the disorders that were retained, an additional search was conducted to find additional papers on zebrafish models, using the names of the diseases and ‘zebrafish’ as keywords.

## 3. A Brief Overview of the Normal Development of the Axial Skeleton in Zebrafish

The development of the teleost skeletal system is a complex spatiotemporally regulated process that—although very similar to that for amniotes—has several distinct characteristics [9,20,21,22,23,24,25,26,27,28,29]. These include the facts that osteocytes are not present in all bones, that certain structures such as the skull show greater complexity compared to mammals, and that many different bone and cartilage types with different cellularity and matrix compositions exist in physiological conditions while often only occurring in diseases in vertebrates [30,31]. For a detailed review of the bone development in zebrafish, we would like to refer the reader to several excellent reviews [4,5,9,20,27,31,32,33,34,35,36]. Here, we will focus on the development of the axial skeleton of teleosts.

**Figure 1:**
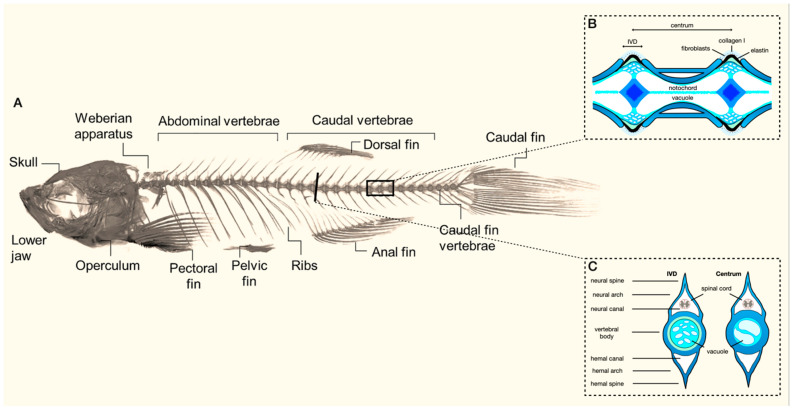
(**A**) Overview of the composition of the axial skeleton of adult zebrafish (modified from Dietrich et al. (2021)) [20]. Inserts illustrate a sagittal (**B**) and transversal (**C**) view of the morphology of the vertebral body and the intervertebral disc (IVD) (adapted from Cotti et al. (2022)) [21].

As part of the endoskeleton of zebrafish, the axial skeleton consists of the vertebral column, ribs, intermuscular bones, and unpaired fins (Figure 1) [4,20]. The vertebral column contains 31 vertebrae in total: 4 vertebrae that form the Weberian apparatus, 10 abdominal vertebrae, 14 caudal vertebrae, and 3 caudal fin vertebrae [20]. The vertebrae—each containing a centrum, hemal and neural arches, and a spine—are connected by a ring-shaped ligament built up from intervertebral soft tissue [3,20]. The developmental timing and origin of the skeletal elements in the axial skeleton are complex; we would like to refer the reader to the work of Bird et al. [4] for a detailed overview, but we touch on a few essential principles below.

Physiological ossification starts around 3 days post fertilization (dpf), beginning with the cranial cartilage. At the same time, the cleithrum, fifth branchial arch, and opercle form through dermal ossification [2,37]. Next, the centra of the axial skeleton are formed, starting with the third and fourth centrums at around 6 dpf [2,4,6,37]. Additional centra are then added bidirectionally [4]. Contrary to the other centra and unlike other fish, the Weberian centra remain unfused throughout development in zebrafish [4].

In contrast to humans, initial vertebral column development in zebrafish is not derived from the sclerotome but takes place through the direct mineralization of the notochord [23,38,39,40,41]. The notochord is composed of chordocytes, which provide a hydrostatic core, and an outer epithelial layer of chordoblasts. The chordoblasts secrete a collagenous matrix during early development that builds the notochord sheath and surrounds the notochord. The segmental mineralization of the notochord sheath induced by BMP and retinoid acid signals leads to the construction of the chordacentrum of the vertebral body [41,42]. Subsequently, sclerotome-derived cells form bone around the chordacentrum, creating the autocentrum.

Though originating from different cell types, it is noteworthy that this process in the vertebral column of teleosts is very similar to what is seen in typical intramembranous bone formation, in which mesenchymal cells condense and differentiate into osteoblasts. This process is initiated by the gene expression of *RUNX2*, which is involved in osteoblast differentiation; its orthologues in zebrafish, *runx2a* and *runx2b,* show 86% amino acid sequence conservation with mammals [37,43]. RUNX2 interacts with bone morphogenetic proteins (BMPs), which initiates osteoblast differentiation and activates genes such as *BGLAP* and *SPP1*, both involved in the deposition of the bone extracellular matrix [37]. The osteoblasts will produce an osteoid, uncalcified bone matrix consisting of collagen type I and osteocalcin, which subsequently mineralize via the precipitation of hydroxyapatite (HA) upon the removal of the mineralization inhibitor inorganic pyrophosphate (PPi) via the enzyme alkaline phosphatase, secreted by the osteoblasts [3,44,45,46].

## 4. Pseudoxanthoma Elasticum

Pseudoxanthoma elasticum (PXE; OMIM# 264800) is an autosomal recessive metabolic disease caused by loss-of-function pathogenic variants in the *ABCC6* (ATP-binding cassette, subfamily C, member 6) gene and—to a much lesser extent—the *ENPP1* (ectonucleotide pyrophosphatase/phosphodiesterase 1) gene [47,48]. In humans, *ABCC6* encodes an ATP-dependent ABC transporter, ABCC6, which is mainly present in the liver and kidneys [49]. Though its substrates remain elusive, it was shown to be indirectly involved in ATP homeostasis, as ABCC6 deficiency was associated with decreased ATP efflux [50]. Circulatory ATP is then metabolized into AMP and PPi by the ENPP1 enzyme [51]. Due to ABCC6 or ENPP1 deficiency, progressive mineralization and fragmentation of the elastic fibers can occur, which results in skin, ophthalmological, and cardiovascular symptoms in PXE patients [52].

**Figure 2:**
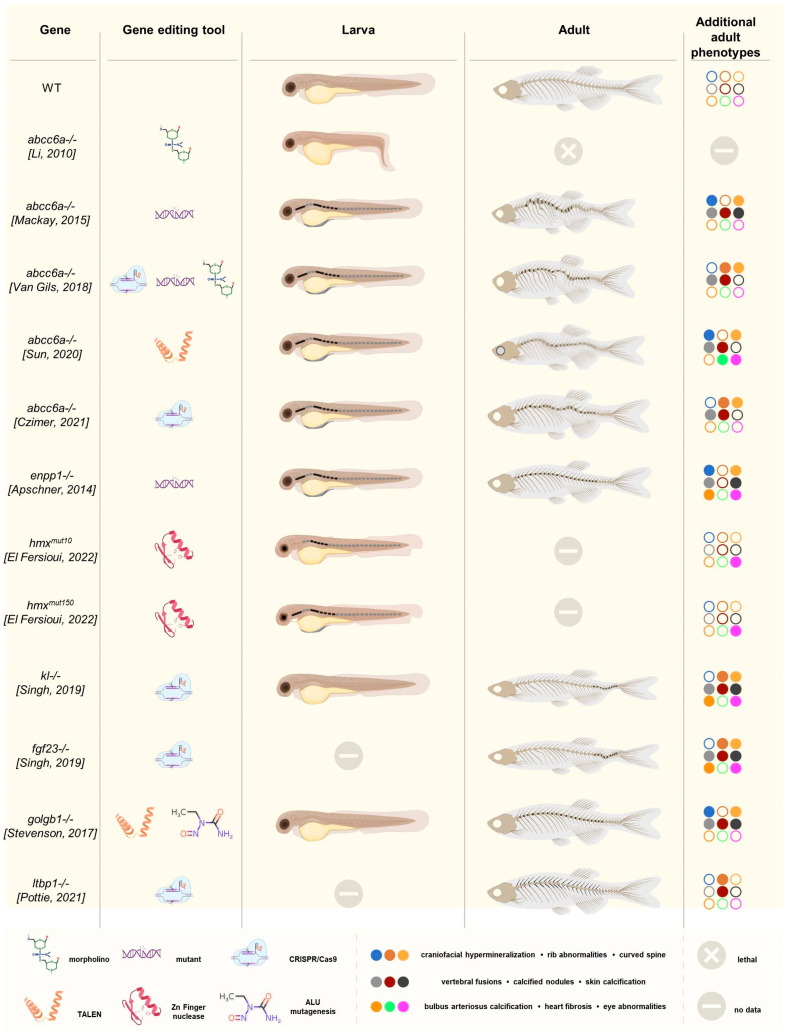
Graphical overview of the discussed zebrafish models, including the targeted gene, the genome-editing tool(s) used, and a schematic representation of the larval and adult ectopic mineralization phenotype (indicated in black). All PXE/GACI models showed advanced vertebral mineralization in the larvae [55,57,58,59,60], except for the model developed by Li et al [53]. In the adult models, excessive (inter)vertebral calcification was observed. Similarly, excessive vertebral mineralization was seen in the *hmx1−/−* larvae [37]. This recurring phenotype was not present in the *klotho−/−* or golgb1−/− larvae, but the adult fish showed extensive vertebral mineralization [61,62]. This was also observed in the adult *ltbp1*-knockout fish, including calcification of the ribs [63]. Finally, the color scheme indicates the similarities and differences between the different adult models in terms of more detailed phenotypic characteristics such as craniofacial hypermineralization, rib abnormalities, the presence of a curved spine, vertebral fusions and calcified nodules on the vertebrae and/or ribs, skin and bulbus arteriosus calcification, heart fibrosis, and eye abnormalities. Filled circles indicate that the corresponding phenotypic feature is present.

Two orthologs of *ABCC6* have been described in zebrafish, *abcc6a* and *abcc6b* [50]. There are several reports that describe the effects of abcc6a deficiency caused by morpholino knock-down or a CRISPR/Cas9-mediated knockout of *abcc6a*. In 2010, Li et al. reported that abcc6a was required for normal zebrafish development [53]. The morpholino-mediated knock-down of the *abcc6a* gene revealed a shortening of the body, delayed development of the head, decreased tail length, and curving of the caudal part of the morphants at 1 dpf (Figure 2). At 3 dpf, cardiac edema was noted, which eventually led to demise at 8 dpf. It must be noted that many of these changes are rather unspecific and frequently encountered in morpholino-edited fish in a dose-dependent manner [53,54,55,56]. Also, the authors did not provide information on aberrant mineralization in their model [53]. In the same study, the knockdown of *abcc6b* did not lead to an aberrant phenotype. Via in situ hybridization, the authors reported that *abcc6a* was expressed in forerunner cells upon gastrulation and then in the Kupffer’s vesicle (which derives from forerunner cells) as well as in the tail bud, while *abcc6b* was mainly expressed in the anterior part of the embryonic kidney proximal straight tubule [53].

Mackay et al. described a gräte mutant *abcc6a* zebrafish harboring a missense substitution p.(L1429R) in the second nucleotide-binding domain (NBD2) [57]. At 8 dpf, hypermineralization along the vertebral column was noted, which lead to a curved spine and reduced length (Figure 2). They also noted enhanced mineralization of craniofacial elements and skin mineralization, though the latter was rare. Mineralization of the vertebrae proceeded faster than in the wild type, leading to vertebral fusions. At 6 weeks post fertilization (wpf), mineralized nodules were observed on the margins of the intervertebral space along the thickened, curved spine. In situ hybridization at 5 dpf showed abcc6a expression in regions of developing bone but not in the liver or kidney. Using reporter constructs, the authors observed the co-expression of osterix and, to some extent, osteocalcin with abcc6a starting at 4 dpf in the operculum and cleithrum. At 20 dpf, *abcc6a* was mainly expressed in the intervertebral disc regions. Based on this, Mackay et al. believed *abcc6a* to be expressed in a population of mature osteoblasts, which is not the case for human *ABCC6*. Interestingly, *abcc6b* expression was confined to the operculum, parasphenoid, and ear cartilage [57].

In 2018, the first CRISPR/Cas9-edited *abcc6a* zebrafish model was described, the editing of which entailed the introduction of a four-base-pair deletion in exon 2, c.180delTCGG [55]. At 10 dpf, advanced mineralization of the vertebrae was seen (Figure 2). At 4.5 and 7.5 months post fertilization (mpf), the fish were shorter, had undergone hypermineralization as well as fusion of vertebral bodies, and developed calcified nodular lesions in the intervertebral spaces and broader and bifid ribs. A similar phenotype was also found in an *abcc6a* splice-site mutant (c.2250+1G>A) and—in the larval stage—in a morphant model. In contrast, no calcification of the eyes or heart was seen at 13.5 mpf. Van Gils et al. did not investigate the abcc6a expression pattern [55].

A fourth model, where *abcc6a* knockout was achieved by introducing 8 bp and 17 bp deletions leading to a truncated abcc6a protein via TALEN, was reported by Sun et al. [58]. The mutants were indistinguishable from the WT at 4 dpf, but at young-adult stages, they were shorter and more curved, with fusions of the vertebral bodies and intervertebral spaces (Figure 2). Also, calcified nodular lesions in the vertebrae were seen; quantification analysis of bone volume and BMD showed increased mineralization in the vertebrae and suborbital and supraorbital bones. Contrary to the previous models, the authors also found fibrosis in the heart and sclera, ocular calcifications, and electron-dense material in the thickened mutant Bruch’s membrane of the eye. In early embryonic stages, *abcc6a* was expressed ubiquitously; at 48 h post fertilization (hpf) and 4 dpf, expression was noted in the heart, operculum, and cleithrum as well as in the ear and notochord [58].

Finally, Czimer et al. used CRISPR/Cas9 editing to create frameshift alleles, p.Arg60Serfs*183 and p.Cys205Leufs*4, in the *abcc6a* and *abcc6b* genes, respectively, both leading to a premature termination codon [59]. *Abcc6a−/−* larvae showed advanced vertebral calcification in both larvae and adult fish, contrary to what was observed for *abcc6b* mutants, and these findings were similar to those reported by MacKay et al. and Van Gils et al. (Figure 2) [55,57]. No gene expression analyses were performed [59].

## 5. Generalized Arterial Calcification of Infancy

Mutations in the *ENPP1* gene were first described in generalized arterial calcification of infancy (GACI, OMIM #208000, #614473), an autosomal recessive disorder characterized by extensive vascular calcification, resulting in cardiovascular complications such as heart failure, respiratory distress, edema, cyanosis, hypertension, and cardiomegaly [64]. Other possible manifestations in the skin and eyes are similar to PXE, and periarticular calcifications, hearing loss, and the development of rickets after infancy have also been reported [64].

More recently, Nitschke et al. discovered that some GACI patients harbor *ABCC6* pathogenic variants, further emphasizing that PXE and GACI are part of one spectrum [47]. Interestingly, an identical *ABCC6* genotype can lead to PXE in one patient and GACI in another, suggesting that other (epi)genetic factors must be at play in determining the definitive phenotype [47].

*ENPP1* has but one orthologue in zebrafish and the *enpp1* mutant, coined *dragonfish* (dgf), which recapitulates features of both GACI and PXE, as they undergo ectopic calcification in soft tissues such as the skin, cartilage, heart, and intracranial space as well as perichondral ossification as early as 4 dpf in some (Figure 2) [60]. Further, hypermineralization of the vertebral column with fusion of vertebral centra occurred in juvenile and young adult fish, together with the calcification of the heart and its outflow (bulbus arteriosus). Of interest and contrary to what is observed in some *abcc6a* zebrafish, these calcifications occur independently of the expression of typical osteoblast or cartilage markers such as osterix and have been suggested to result from passive calcium deposition [60].

The ubiquitous expression of *enpp1* was noted, with some of the highest expression levels observed in bone elements, such as the operculum and cleithrum, similar to the expression pattern in mice. Besides the local function of the phosphodiesterase, it was shown that *enpp1* also acts in areas that are remote from its expression site, probably reflecting the anti-mineralizing effect of PPi [60].

## 6. Schorderet–Munier–Franceschetti Syndrome

Also known as oculoauricular syndrome (OAS; OMIM#612109), Schorderet–Munier–Franceschetti syndrome is a rare autosomal recessive disease that has been described in two consanguineous families [65,66]. It is characterized by complex ophthalmological and external ear anomalies as well as aberrant orofacial development with underdeveloped and asymmetric jaws [37]. OAS is caused by homozygous pathogenic variants in *HMX1* (H6 Family Homeobox 1), encoding a homeobox transcription factor with high affinity toward a consensus HMX-binding site harboring a 5′-CAAGTG-3′ element required for sensory organ development [65,66,67,68]. *HMX1* is highly conserved across orthologs and contains three essential domains: a homeobox domain (HD) and conserved domains SD1 and SD2 [37,65,66]. HD and SD1 are involved in the dimerization of HMX1, while the function of SD2 remains unknown.

Two zebrafish mutants have been generated to ascertain the OAS phenotype using zinc-finger nucleases that target two different conserved regions of the orthologue *hmx1* gene [37]. *Hmx1^mut10^* zebrafish harbor a frameshift variant resulting in a premature termination codon in the SD1 domain, while *hmx1^mut150^* zebrafish have an indel in the HD domain that obliterates this domain’s function. Both mutants prevent the dimerization of SD1 and HD—an essential prerequisite for proper hmx1 function—leading to a loss-of-function effect [37]. *Hmx1* is mainly expressed during the development of the eyes and ears, starting at the 10-somite stage, which correlates with 14 hpf [33,69]. The mutant zebrafish developed smaller eyes and presented with delayed neurogenesis paired with increased apoptosis in the retina and brain [68,70]. Interestingly, *hmx1* knockdown resulted in defects in regions where *hmx1* is not expressed, such as the vertebral column [70]. Both mutants presented normal cartilage and bone development until 5 dpf, but premature mineralization was observed from day 7 onward (Figure 2) [37]. After this point, 70% of the wild-type zebrafish at 8 dpf showed one mineralized vertebra, while 80% of the *hmx1^mut10^* and *hmx1^mut150^* zebrafish presented with five mineralized vertebrae [37].

To explain the advanced mineralization phenotype, a dysregulation of the BMP gradient that regulates dorsal/ventral patterning in zebrafish was put forward [71]. Physiologically, the concentration of BMP is highest ventrally and is inhibited dorsally by antagonists such as chordin, noggin, and follistatin, which play essential roles in the formation of the axial skeleton [37,71,72,73,74]. In *hmx1* mutants, reduced RNA levels of *chordin* and *noggin1* result in a pro-osteogenic environment with increased expression levels of *bmp2b* and *bmp4b* and the subsequent upregulation of osteogenic markers *runx2b* and *spp1* [37]. *Runx2b* initiates endochondral and intramembranous ossification, and its complex with BMP initiates osteoblast differentiation. The treatment of the mutants with the selective BMP inhibitor DMH1 (dorsomorphin homolog 1) restricted vertebral mineralization, indicating that this mineralization is indeed BMP-driven, possibly resulting from increased *bmp2b* and *bmp4b* activity [37]. While it is unlikely that *hmx1* directly regulates *bmp2b* and *bmp4b* since they do not have the appropriate CAAGTG binding element, it is possible that *hmx1* is involved in the regulation of *noggin1* and *chordin* expression, as the promotor regions of these BMP inhibitors contain *hmx1* binding sites, but there is currently no experimental evidence to confirm this hypothesis [37]. Another known *hmx1* target with a regulatory function is *uhrf1* (Ubiquitin-Like With PHD and Ring Finger Domains 1), a multi-domain key epigenetic regulator protein involved in chromatin modifications and cellular proliferation. *Hmx1* was shown to inhibit *uhrf1* in mutant zebrafish, though this effect was mainly limited to the cranial region and therefore seems less likely to be involved [37,68].

## 7. Hyperphosphatemic Familial Tumoral Calcinosis

Hyperphosphatemic familial tumoral calcinosis (HFTC, OMIM# 211900, 617993, 617994) is an autosomal recessive metabolic disorder characterized by the progressive deposition of calcium phosphate crystals in periarticular soft tissues, particularly after repetitive trauma or pressure, and painful swellings [75]. Dental anomalies and—less frequently—vascular, testicular, and retinal calcifications can also occur. The biochemical hallmark of tumoral calcinosis, hyperphosphatemia, is caused by increased renal absorption of phosphate due to pathogenic variants in the *FGF23*, *KLOTHO* (*KL*), or *GALNT3* genes. Under physiological conditions, serum calcium and phosphate concentrations are strictly regulated through hormonal mechanisms via the coordinated action of parathyroid hormone (PTH), vitamin D, and fibroblast growth factor-23 (FGF23) [76,77]. FGF23 is a hormone that downregulates renal phosphate reabsorption and promotes urinary phosphate excretion [45,78]. αKlotho is a single-pass transmembrane protein encoded by the *KL* gene, which is predominantly expressed in the distal convoluted tubules of the kidney where it binds to FGF receptors and functions as an FGF23 co-receptor [78,79]. The *GALNT3* gene encodes for the enzyme N-acetylgalactosaminyltransferase 3, responsible for the O-glycosylation of FGF23 [80,81,82,83]. The absence of this post-translational modification leads to poor FGF23 secretion. In humans, FGF23 is secreted by osteocytes in bone, but in zebrafish, it originates from teleost-specific kidney-associated glands, namely, the corpuscles of Stannius, and from the gills [61,78]. Even though the origins of this hormone are different, the zebrafish *fgf23* gene is evolutionary conserved, and its function in maintaining mineral homeostasis has been preserved [61,84]. This is also true for the *kl* gene, which is expressed in the brain, pancreas, liver, and kidneys in embryonic and larval zebrafish but only in the liver and kidney in adults [61,84].

α*Klotho* and *fgf23* CRISPR/Cas9-mediated knockout zebrafish were reported to have an indistinguishable phenotype from each other, with adult-onset morbidity and mortality, starting at 4 to 5 months of age, with loss of fin integrity, eye overgrowth, widespread ectopic calcification (especially in the outflow tract of the heart but also in the vasculature of the dermis and muscles), and spinal deformities (Figure 2) [61,78]. The last features were most prominent in the caudal region, with multifocal areas of hyperostosis and chondrodysplasia. These regions of bone overgrowth were accompanied by areas of dystrophic calcification, connective tissue proliferation, and immune cell infiltration [61,78].

There have been no reports on *galnt3* mutant zebrafish to date, and little is known about its expression sites. However, a near-complete loss of function of *galnt3* was observed in the bone of zebrafish knockout models of giantin, a golgin protein (encoded by the *golgb1* gene) important for Golgi organization and vesicular transport [62]. Two *golgb1* mutants with a premature stop codon mutation due to ENU mutagenesis and TALEN site-directed mutagenesis, respectively, did not display any gross developmental defects and had normal lifespans but presented ectopic mineralization of soft tissues in adulthood. Specifically, in the axial skeleton, hyperostosis and ectopic mineralization of the intervertebral discs were seen, leading to a reduction in vertebral spacing and vertebral fusion as well as ectopic calcified deposits in multiple vertebrae [62].

Mechanistically, the greatest amount of attention has been paid to vascular calcification in the bulbus arteriosus of *klotho* and *fgf23* mutants, where an osteogenic gene expression profile was observed in the heart and vascular smooth muscle cells, with increased expression of *mmp9*, *mmp13*, *spp1*, and, more modestly, *runx2b* [61,85]. Besides pathways involved in bone formation and remodeling, inflammatory pathways were also upregulated in the *kl−/−* mutants. More recently, it was proposed that cellular senescence contributes to the ectopic calcification and premature death of these mutants, opening the possibility of using these mutants to model (premature) aging [86,87,88].

## 8. LTBP1-Related Cutis Laxa Syndrome

Cutis laxa (CL) comprises a large group of inherited and acquired diseases characterized by reduced elastic recoil leading to a loose, saggy, and aged appearance [89]. The inherited forms of CL (OMIM# 150390) can be autosomal dominant or recessive and are induced by pathogenic variants in a variety of genes, including *ALDH18A1*, *ATP6V0A2*, *ATP6V1E1*, *ATP7A*, *EFEMP2*, *ELN*, *FBLN5*, *GORAB*, *LTBP1*, MAP kinases, *PYCR1*, and *RIN2*. Among the CL zebrafish models that were generated, only *ltbp* mutants displayed an axial skeletal phenotype [63].

LTBPs (Latent transforming growth factor β (TGF-β)-binding proteins) are microfibril-associated extracellular matrix (ECM) proteins that anchor TGF-β onto the ECM and regulate its storage, release, and activation [63,90]. They are also responsible for the correct assembly of ECM proteins and are considered structural components of connective tissue [63,90]. In patients, bi-allelic *LTBP1* pathogenic variants cause an autosomal recessive CL syndrome featuring cutis laxa, facial dysmorphism, cardiac defects, and skeletal abnormalities such as short stature and craniosynostosis [63].

Human LTBP1 has two isoforms: the long isoform (LTBP1L) is essential for TGF-β signaling and cardiovascular development, while the short isoform (LTBP1S) might play a role in craniofacial development [63]. This protein is both localized in the ECM, where it interacts with fibrillin-1 and fibronectin via its C- and N-terminal regions, respectively, [63], and in newly forming bone (osteoid), illustrating that LTBP1 might play a role in bone and connective tissue [90].

In contrast to humans, zebrafish only have one highly conserved long isoform of ltbp1, which is expressed in the bulbus arteriosus, pancreas, brain, liver, and cranial bones [63,90,91]. Several models have been reported, among which number *ltbp1−/−* Δ29 (c.3526del) and *ltbp1−/−* Δ35(c.4294_4303del), which were generated using CRISPR/Cas9 and harbor a premature stop codon [63]. Both mutants had normal viability but showed a significant increase in ectopic bone formation of intramembranous origin on the neural and hemal arches of the vertebrae at 4 mpf, especially at the base of the arches (Figure 2). This was accompanied by decreased tissue mineral density in both vertebrae as well as vertebral and hemal arches in the *ltbp1−/−* Δ29 mutant but not in the *ltbp1−/−* Δ35 mutant. Additionally, bone volume and thickness were increased in the *ltbp1−/−* Δ29 mutant only, possibly because the C-terminal TGF-β-binding domains are only affected in this mutant [63].

For the two other *ltbp1* zebrafish models, a morphant knock-down and a TALEN-induced knockout, respectively, skeletal mineralization was not investigated, and the models were only evaluated as larvae [90,91]. Xiong et al. described the morphants as being shorter, so an axial phenotype might have remained undetected [90]. It is hypothesized that the collagen maturation defect that was seen in *ltbp1* mutants contributes to ectopic bone formation, but no experimental evidence is available to date to confirm or refute this hypothesis [63].

## 9. Discussion

The zebrafish has been an outstanding research organism for studying molecular mechanisms, cellular signaling, and the treatment possibilities for a wide variety of diseases [92]. Among these, the axial skeleton has proven to be very useful for modeling diseases that primarily affect the vertebral column in humans, such as scoliosis and osteoporosis [23,93,94]. Further, zebrafish models for various bone disorders, such as osteogenesis imperfecta and fibrodysplasia ossificans progressive (FOP), feature abnormalities of the axial skeleton, and their respective causal genes usually have a well-documented role in bone development and repair (Table 1) [8,95,96,97,98,99,100,101,102,103,104,105]. Remarkably, the zebrafish models that are discussed in this review feature a striking axial skeletal phenotype, while the human disease they model does not show vertebral column abnormalities, even though the human phenotype affects multiple organ systems.

For some of these disorders, such as PXE or GACI, the vertebral phenotype is the main—if not the only—feature of the zebrafish mutants and knockouts [53,55,57,58,59,60]. PXE is considered a hallmark disease with respect to studying ectopic soft tissue mineralization, c.q., hydroxyapatite precipitation, occurring in several genetic and acquired diseases as well as during aging [50,60,106]. As a multisystemic disorder, PXE affects several organs, but the vertebrae remain unaltered. Indeed, bone development and morphology have been studied in PXE patients and were found to be normal, except for a potentially higher propensity for osteoarthritis in the knees and acromioclavicular joints [107,108]. A variety of zebrafish models have been created for PXE, and though differences exist—regardless of whether they related to the underlying genetic defect or genome-editing technology—the vertebral phenotype is rather consistent and can therefore be considered a true consequence of *abcc6a* deficiency [53,55,57,58,59,60]. Several groups have demonstrated that this phenotype is readily amendable to experimental perturbation via drugs using vitamin K as an example, enabling its use as a screening read-out for compounds such as bisphosphonates, sodium thiosulphate, magnesium citrate, and the PARP inhibitor minocycline [57,58,109,110]. Except for vitamin K, the effects of the evaluated drugs were similar to what was observed in PXE mouse models or patients, proving that zebrafish are a good preclinical intermediate model for anti-mineralizing drug screening [111]. Zebrafish models can be used after in vitro experiments to screen and select compounds that may target (ectopic) calcification before evaluating a drug’s effect in other vertebrates such as rodents [57,58,109,110]. The results obtained with vitamin K, which could not be replicated in mice, however, indicate that screening results for teleosts are not always applicable to vertebrates and highlight why rodent experiments—though they can be reduced in number—remain necessary [112,113]. Similarly, drug interventions were also conducted in the *enpp1* and *hmx1* models using axial skeleton mineralization as a read-out, although more modestly than in *abcc6a* mutants [37,60]. Altogether, these first pharmacological experiments confirm the great potential of zebrafish in drug discovery and evaluation for the treatment of multisystemic diseases. The relatively easy and robust evaluation and quantification of a vertebral phenotype, both in larvae and adult fish, add to the great potential of these animal models. In addition, it is possible to study drug activity at single-cell resolution within the complexity of an entire animal, across tissues and over an extended timescale, when combined with cell-specific or tissue-specific reporters and gene-editing technologies [114].

**Figure 3:**
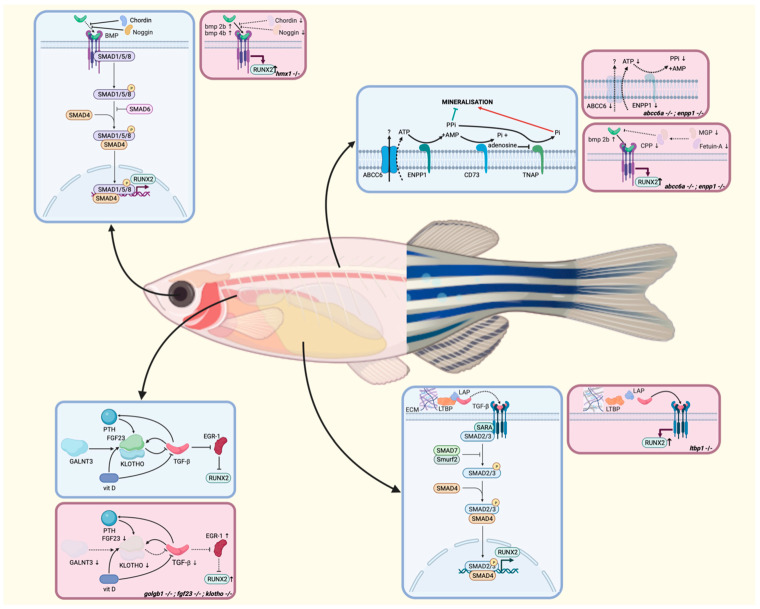
Signaling pathways involved in ectopic mineralization in zebrafish in physiological circumstances (blue) and disease (pink) for each of the discussed models, depicted at the site of highest expression for each gene. In the pathophysiological illustrations, arrows indicate increased or decreased expression of the involved mediators. AMP: adenosine monophosphate; ATP: adenosine triphosphate; BMP: bone morphogenetic protein; CPP: calciprotein particle; ECM: extracellular matrix; LAP: latency-associated peptide; LTBP: latent transforming growth factor *β*-binding proteins; PTH: parathyroid hormone; P: phosphorylated; Pi: inorganic phosphate; PPi: inorganic pyrophosphate; SARA: smad anchor for receptor activation; vit D: vitamin D. Adapted from [37,90,128,129,138,152,153,155].

A challenge for most of the models addressed in this review was attaining a comprehensive understanding of how the axial skeleton phenotype occurs. When looking at the sites of expression of the different genes and proteins involved, most are not expressed at the sites of hypermineralization. *Abcc6a* may be an exception, as it was suggested to be expressed in osteoblasts in zebrafish, though discrepancies exist between different studies [53,57,58]. This proposed expression pattern completely differs from that of human and murine *ABCC6*, which is mainly expressed in the liver and kidneys, though species-, population-, and context-specific expression is not unusual [49,115]. Interestingly, in the *Abcc6*−/− mouse model, Boneski et al. and Kauffenstein et al. independently revealed the presence of progressive vertebral osteopenia but not intervertebral disc or other axial ectopic calcifications. This murine phenotype is essentially characterized by trabecular bone loss and mainly occurs in older knockout mice. The authors suggested it to be closely linked to increased osteoclastic activity, but, at the same time, these observations pose the question of the importance of PPi/Pi balance in bone homeostasis (Figure 3) [111,116]. Moreover, the ABCC6 transporter facilitates ATP efflux, and PXE patients have lowered plasma levels of PPi compared to healthy controls and heterozygous carriers [49,117,118,119,120]. The existence of a subset of adult GACI patients with pathogenic variants in *ABCC6* or *ENPP1* that have a complete lack of PPi in their plasma and suffer from hypophosphatemic rickets strengthens the belief that PPi/Pi balance may have an influence on bone, though there is currently little additional experimental evidence for this hypothesis [121]. The *abcc6a* and *enpp1* zebrafish provide excellent opportunities for studying the roles of ABCC6 and ENPP1, their downstream signal transduction proteins, and PPi in bone since they are required for the generation of PPi [53]. As the effects on bone may be the most impactful over time, further study of the vertebral phenotype in these models can expand our knowledge about (bone) aging and how it can be linked to cellular senescence. Senescence plays a role in childhood bone-growth-associated bone mass acquisition and contributes to the development of osteoarthritis and osteoporosis, but a detailed characterization taking into account the heterogeneity of senescence-related signaling and differences depending on cell type and senescence-inducing stimuli remains elusive [122,123]. *Abcc6*, but also *fgf23* and *kl*, have been previously associated with downstream senescence signaling pathways, and their respective models may therefore hold answers to some of these outstanding questions [124,125,126,127].

As for the other genes discussed in this paper, their expression profiles resemble those of their human counterparts, and their downstream signaling mediators—TGF-β and BMP-RUNX2—have established roles in bone formation and homeostasis (Figure 3) [128]. In physiological circumstances, TGF-β and BMP signal through a canonical SMAD pathway and a noncanonical SMAD-independent pathway [129,130,131,132,133]. Both pathways lead to the expression of osteogenic transcription factors such as RUNX2 and regulate mesenchymal stem cell differentiation, bone formation, and bone homeostasis [128,129,134]. Continuous activation of the BMP pathway leads to ectopic bone formation, as illustrated by FOP, caused by gain-of-function pathogenic variants in the type 1 BMP receptor (encoded by the *ACVR1* gene) [95,135,136,137]. Furthermore, inhibitors of BMP2 signaling, such as matrix gla protein (MGP) and fetuin-A, also play a role in maintaining the balance between a pro- and anti-osteogenic environment. By binding together in calciprotein particles (CPP), they capture hydroxyapatite in body fluids [138,139,140,141,142]. These so-called primary CPPs are initially spherical, amorphous, and soft but can spontaneously transform into secondary CPPs that are larger, crystalline, and less soluble [143,144]. The intricate relations between these pro- and anti-calcification mediators have been well-documented in, e.g., murine and human PXE, with low serum levels of MGP and Fetuin-A, contributing to the upregulation of *RUNX2* through the BMP2-SMAD-RUNX2 and TGFB2-SMAD2/3 pathways [128,138,145,146,147,148] but also affecting the calcification propensity T50 test, which reflects the time required to convert 50% of primary CPPs in a serum sample into secondary CPPs [149,150,151]. The latter was significantly prolonged and associated with disease severity in PXE [146]. In the two *hmx1* mutants for Schorderet–Munier–Franceschetti syndrome, bmp also plays a crucial role, as loss of inhibition via *chordin* and *noggin1* creates a pro-osteogenic environment with increased expression of *bmp2b* and *bmp4b* and upregulation of *runx2b*, initiating mineralization through endochondral and intramembranous ossification and osteoblast differentiation [37,152,153,154,155]. Conversely, TGF-β plays a central role in the pathophysiology of both hyperphosphatemic tumoral calcinosis and the LTBP1-related CL syndrome. In the former, the complex interplay between TGF-β and FGF23, influencing each other directly and indirectly via the co-receptor Klotho and the active vitamin D metabolite 1,25(OH)2D3 in a regulatory feedback mechanism, results in osteoblast proliferation [156,157,158,159,160,161,162,163,164,165]. Additionally, mouse studies have revealed that Klotho indirectly inhibits *Egr-1* expression, a transcription factor involved in several signaling pathways such as apoptosis and angiogenesis, by inhibiting the TGF-β pathway [166,167]. This transcription factor stimulates osteogenic differentiation and upregulates osteogenic markers such as *Runx2* and *Sox9* [168]. Klotho-deficient mice present an upregulation of the TGF-β pathway, followed by upregulated *Egr-1*, *Runx2*, and *Sox9* as a result. The LTBP1-related CL syndrome reduces the anchorage of TGF-β in the ECM, leading to the continuous activation of the TGF-β signaling pathway and, again, *Runx2* expression [63,169,170].

This documented involvement of ubiquitously active key mineralization mediators brings us to a final intriguing, outstanding question: why is only the vertebral skeleton affected in these models? Such compartmentalized effects suggest the existence of tissue-specific regulatory mechanisms of mineralization. While it is a well-documented concept that the mineralization process is unique to mineralized tissues, e.g., bone versus dentine or enamel, the mechanisms underlying the diversity of mineralization within a specific tissue are less well described. In bone, the unravelment of the complex mixture of heterogeneous cell types has only just begun; many discrete populations, e.g., mesenchymal cells, seem to exist, the dynamics of which are influenced by external stimuli and impact bone formation [114]. Such compartmentalization is also seen in pathological mineralization in hereditary or acquired diseases and aging, e.g., in the selective elastic fiber mineralization in PXE, but the underlying mechanisms remain largely unknown. Again, this makes the zebrafish strains discussed in this paper outstanding models with which to gain insights into the tissue- and cell-specific regulatory mechanisms of mineralization via, e.g., the use of single-cell transcriptomic profiling.

**Table 1:** Zebrafish models for bone disorders with a vertebral phenotype.

Disease	Human Gene	Zebrafish Orthologue	Knockout Vertebral Phenotype	References
Bruck Syndrome type II	*PLOD2*	*plod2*	Shortened body axis, kyphoscoliosis, compressed vertebrae, excess bone at vertebral end plates that results in loss of hourglass shape of vertebra, increased TMD and thickness	[8]
FOP	*ACVR1*	*acvr1l*	Dorsalization of the embryonic axis	[95,96,97]
Craniosynostosis	*CYP26B1*	*cyp26b1*	Outgrowth of cartilaginous endochondral disc in pectoral fins, coronal craniosynostosis	[171,172]
Osteoporosis	*ATP6V1H*	*atp6v1h*	Premature death, reduction in/absence of bone cells, almost complete absence of mineralized bone	[94]
Osteogenesis imperfecta	*COL1A1* *COL1A2* *BMP1* *SP7*	*col1a1a, col1a1b* *col1a2* *bmp1a, bmp1b* *sp7*	Variable phenotype including callus formation, bending of ribs, short stature, craniofacial abnormalities, malformation of vertebral column	[8,99,100,101,102,103,104,105]
Ehlers-Danlos syndrome	*B4GALT7*	*b4galt7*	Scoliosis; small, bent pectoral fins; reduced or absent mineralized bone	[10,173]
Spinal curvature disorders	*COL8A1* *KIF6* *PTK7* *TBX6*	*col8a1a, col8a1b* *kif6* *ptk7a* *tbx6*	Extensive scoliosis in thoracic and caudal parts of the spine, deformed and fused vertebrae, fused neural and hemal arches	[10,174,175,176,177]
Gaucher disease	*GBA1*	*gba1*	Reduced osteoblast differentiation and bone mineralization, slight curvature of the trunk	[178]

## 10. Conclusions

The axial skeletal phenotype of zebrafish models offers a good read-out for compound detection and preclinical evaluation in hereditary multisystemic diseases. This clear and great potential of zebrafish should lead to increased efforts to identify and screen drugs for these complex disorders that are currently often intractable. Furthermore, they can be a valuable aid in untangling the mechanisms—in general but also in tissue- and cell-specific cases—that contribute to physiological and pathological mineralization. Aside from the many advantages of using zebrafish as a model system, there are limitations to their use. Besides the fact that not all human genes have a zebrafish orthologue and that parts of their physiology and anatomy are not exact replicas of those in humans, a major drawback is the difficulty of blood drawing, particularly in the field of ectopic mineralization, as circulatory mineralization regulators cannot be reliably evaluated. Further, skeletal development in teleosts is very prone to environmental factors, contrary to the case for mammals. Though zebrafish cannot, therefore, completely replace murine models in modeling and studying human mineralization, they have become an indispensable intermediate model for performing mechanistic studies and targeted and high-throughput drug-screening and pharmaceutical pilot studies, thereby contributing to reducing the use of mammals in biomedical research.
